# From organoid culture to manufacturing: technologies for reproducible and scalable organoid production

**DOI:** 10.1038/s44385-025-00054-6

**Published:** 2026-02-02

**Authors:** Dohui Kim, Jaeseung Youn, Juhyeok Kim, Jeongmin Lee, Jangwon Yoon, Dong Sung Kim

**Affiliations:** 1 Department of Mechanical Engineering, Pohang University of Science and Technology (POSTECH), Pohang, South Korea; 2 Division of Interdisciplinary Bioscience and Bioengineering, Pohang University of Science and Technology (POSTECH), Pohang, South Korea; 3 Graduate School of Convergence Science and Technology, Pohang University of Science and Technology (POSTECH), Pohang, South Korea

**Keywords:** Biological techniques, Biotechnology, Engineering, Medical research, Stem cells

## Abstract

In this review, we systematically categorize diverse organoid engineering strategies—including cellular programming, material engineering, and platform- or system-level innovations—according to their impact on reproducibility and scalability, and highlight representative applications and emerging directions. By reframing organoid generation as a manufacturing process, these technological advances pave the way toward standardized and high-fidelity organoid production for both fundamental research and translational applications.

## Introduction

Organoids are three-dimensional (3D), self-organizing multicellular structures derived from stem cells that replicate key structural and functional features of human organs^1,2^. Over the past decade, robust protocols have been established for a wide range of organ systems and pathological contexts, enabling the generation of physiologically relevant models for investigating human development, disease mechanisms, and therapeutic responses^3–5^. Reflecting their translational potential, regulatory bodies such as the U.S. Food and Drug Administration (FDA), the National Institutes of Health (NIH), and European agencies have recently recognized organoids as promising alternatives to conventional preclinical models^6–8^. Similar efforts toward regulatory standardization are also emerging globally, including recent guidelines on organoid manufacturing and application issued by the Korean Ministry of Food and Drug Safety and the Organoid Standards Initiative^9^. In parallel, organoid-focused biotechnology companies and major pharmaceutical firms are rapidly integrating these platforms into drug discovery pipelines, toxicological assessment, and precision medicine initiatives^10,11^. Together, these advances signify a pivotal shift in organoid research, from an emerging laboratory methodology to an industrial-scale technology with imminent clinical and commercial impact.

As organoid technologies continue to evolve, there is increasing interest in transitioning from biological proofs-of-concept to practical implementation. In this context, persistent challenges related to reproducibility and scalability in organoid production have emerged as major barriers to broader application in research, clinical, and industrial domains^12–14^. Conventionally, organoids have been generated through spontaneous self-organization and culture under minimally controlled environmental conditions^15^. While this bottom-up approach recapitulates key aspects of natural organogenesis, it often leads to significant batch-to-batch variability in organoid architecture due to poorly controlled culture conditions such as matrix composition and spatial organization^16,17^. In addition, conventional organoid culture processes, which are typically carried out using low-throughput platforms and rely on manual handling, offer low production efficiency^18^. These issuesundermines assay fidelity in high-throughput applications and can yield inconsistent biological responses, complicating the evaluation of pharmacological efficacy and safety. Screening platforms generally require large sample sizes to ensure statistical robustness, further emphasizing the need for scalable production systems. In addition, applications in tissue engineering and cell-based therapies demand the generation of large quantities of organoids with reproducible structural and functional characteristics, underscoring the importance of robust, scalable workflows suitable for both research and clinical use.

Despite the absence of a fully established regulatory framework or unified technological standard for industrial- and clinical-grade organoid biomanufacturing yet, substantial progress has been made toward building the technical and institutional infrastructure required for scalability and reproducibility. The Organisation for Economic Co-operation and Development (OECD) introduced the Good In Vitro Method Practices (GIVIMP)^19^, an international quality-assurance framework that defines laboratory quality systems, method qualification, reference controls, equipment calibration, and data integrity—principles that now potentially serve as quantitative benchmarks for process validation in organoid production. Complementing this, the NIH Standardized Organoid Modeling (SOM) Center was recently established to promote the development of organoid platforms that are reproducible, robust, and broadly accessible for translational biomedical and pharmaceutical research.

Expanding these standardization efforts, a recent publication introduced the *Essential Guidelines for Manufacturing and Application of Organoids*, delineating a systematic workflow encompassing cell sourcing, culture optimization, quality control, and biobanking logistics^20^. Their framework identifies organ-specific critical quality attributes (CQAs)—including growth-factor composition, morphological fidelity, and quantitative analytical metrics—and recommends standardized cryopreservation conditions (~100–200 organoids per vial) to enhance batch comparability. Likewise, a recent study established quantitative criteria for human intestinal organoid standardization, specifying cell-line provenance, minimum lineage composition thresholds (e.g., ≥30% enterocytes), and molecular marker expression profiles consistent with physiological differentiation^21^. Taken together, these coordinated initiatives—from international organizations to national agencies and individual laboratories—represent an emerging global framework toward reproducible, quality-controlled, and scalable organoid biomanufacturing, laying the groundwork for eventual regulatory convergence and clinical translation.

In response to these prevailing limitations and in alignment with global standardization trends, a range of engineering strategies has been developed, shifting the paradigm from organoid culture to organoid manufacturing by enabling reproducible and scalable organoid production. These strategies broadly focus on two goals: (1) improving reproducibility by minimizing uncontrolled variation in the culture environment as well as by regulating intrinsic morphogenetic processes, and (2) enhancing scalability by increasing productivity and throughput. To this end, recent advances can be categorized into three major domains: cellular engineering approaches that regulate morphogenetic processes through programmed cell organization; material-based strategies that establish defined and controllable environmental cues; and platform- or system-level innovations that enable high-throughput and automated workflows. Together, these innovative engineering advances mark a transition toward more standardized, efficient production workflows.

**Fig. 1:**
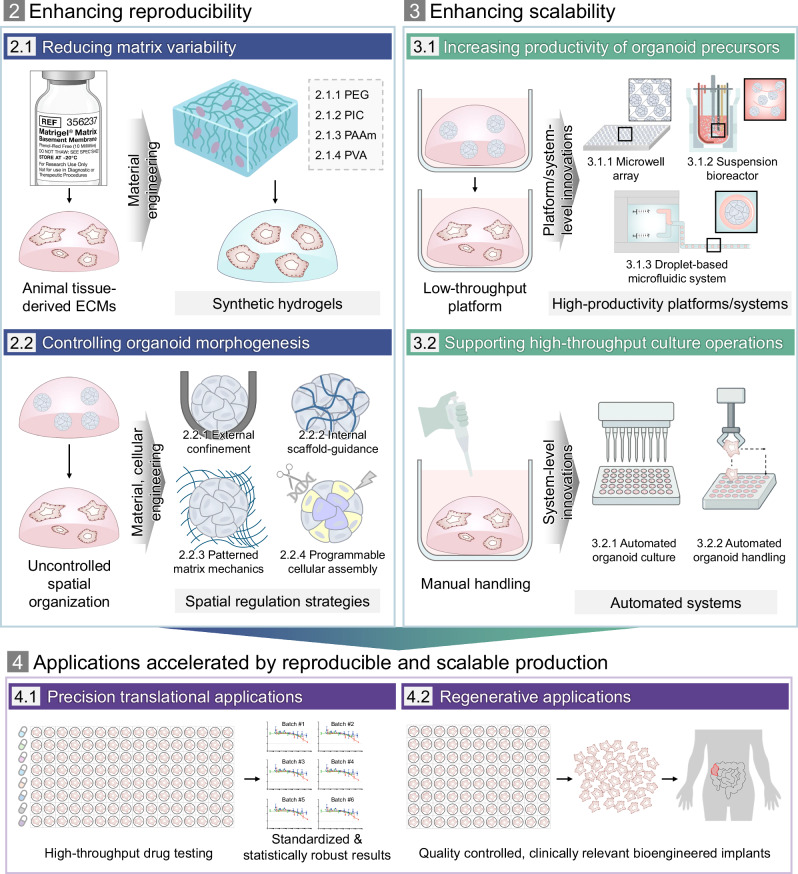
Schematic overview of the major thematic areas discussed in this review. Numbers indicate the corresponding sections.

While several previous review articles have explored bioengineering techniques to improve organoid functionality or have focused on specific biological applications^12,22–25^, few have systematically categorized these engineering strategies in terms of their contributions to enhancing reproducibility and scalability in organoid production. In this review, we provide a focused synthesis of recent advances in organoid bioengineering aimed at addressing these critical challenges (Fig. 1). Specifically, the major barriers to reproducibility stem from the use of biologically variable materials with poorly defined properties, as well as the inherent stochasticity of morphogenetic processes resulting from uncontrolled spatial organization. In terms of scalability, challenges arise from the limited productivity of conventional culture platforms, coupled with the labor-intensive nature of culture processes and handling steps. We then outline engineering strategies developed to overcome each of these challenges and illustrate how they have been applied to improve organoid consistency and yield. Furthermore, we describe representative applications that have leveraged these strategies and discuss emerging directions for advancing organoid production technologies.

## Engineering strategies for enhancing reproducibility

Reproducibility remains a critical barrier to the broader application of organoid technologies, largely due to uncontrolled variation across biological and environmental dimensions of the culture process^22^. Organoids are typically formed through self-organizing developmental programs that are highly sensitive to minor fluctuations in culture conditions^15^. As a result, even minor deviations in culture conditions can lead to significant differences in organoid size, morphology, and functional properties, ultimately undermining consistency across experiments, batches, and laboratories. These variations arise from two primary sources of variability. The first major factor limiting the reproducibility of organoid production is the batch-to-batch variability of animal tissue-derived extracellular matrices (ECMs), such as Matrigel and Geltrex^26,27^. While these matrices are widely used due to their commercial availability and their ability to support organoid growth and morphogenesis, they exhibit substantial inconsistencies in critical properties, including ECM composition, concentration, stiffness, and viscoelasticity^28–30^. Derived from mouse sarcoma tissues, these matrices have an undefined molecular composition and poorly controlled mechanical characteristics, leading to unpredictable cell–matrix interactions. Consequently, these inconsistencies often result in divergent organoid development and morphogenesis. Second, spatial variability arises from uncontrolled geometric, mechanical, and cellular organization during the organoid culture process, often resulting in unregulated tissue expansion or disorganized morphogenesis^15,31^. These sources of heterogeneity are particularly problematic in high-throughput screening, translational research, and clinical manufacturing, where uniformity in organoid structure and function is essential.

In response, a range of engineering strategies has been developed to mitigate these challenges by minimizing matrix-related variability in biochemical and mechanical properties and by guiding organoid morphogenesis with greater precision. In the following sections, we examine these strategies in detail, beginning with approaches aimed at improving matrix definition and consistency.

### Strategies for reducing matrix variability

Organoid culture protocols vary by organ type and developmental stage, encompassing formats such as suspension culture, hydrogel embedding, or hybrid approaches. Despite this diversity, most protocols rely on matrix support, particularly ECM-rich hydrogels, to guide tissue morphogenesis. For example, cerebral cortex organoids have been developed using suspension cultures supplemented with low concentrations of Matrigel^32^. Intestinal organoids are commonly established by embedding stem cells in Matrigel domes^33^. Kidney organoids are either continuously exposed to ECM components like Matrigel and Geltrex^34^ or exposed only during early differentiation stages^35^. While these matrices are effective in supporting organoid growth, their undefined molecular composition, batch-to-batch inconsistency, and poorly controlled mechanical properties introduce significant variability in cell–matrix interactions^36^. This variability often results in heterogeneous organoid size, morphology, and functional readouts, posing a major challenge for reproducibility across experiments, batches, and laboratories^37,38^. Natural hydrogels such as collagen and fibrin can offer improved definition compared to tumor-derived ECMs, but they still retain intrinsic biological variability, limited tunability, and potential immunogenicity^39–42^.

Consequently, increasing attention has been directed toward synthetic hydrogels, which are composed of fully defined polymer networks with precisely controlled chemical and mechanical characteristics. Unlike biologically-derived matrices, these systems exhibit minimal batch-to-batch variability and allow the incorporation of ECM components or adhesive ligands, such as RGD peptides, laminin fragments, or other defined motifs, in a controlled manner. This modularity enables the recapitulation of essential cell–ECM interactions while preserving the reproducibility of the material properties. Among various synthetic hydrogels, polyethylene glycol (PEG), polyisocyanide (PIC), polyacrylamide (PAAm), and polyvinyl alcohol (PVA) have been actively explored for organoid applications (Table 1)^29,43^. Their successful application across multiple organoid types highlights their potential to support controllable and reproducible organoid culture under well-controlled conditions. In the following subsections, we focus on these four hydrogels in detail and describe how their material properties have been engineered to meet the demands of reproducible organoid production.

**Table 1:** Synthetic hydrogels for reproducible organoid production

Hydrogel type	Advantage	Disadvantage	Organoid type	Ref.
PEG	• High commercial availability • Tunable molecular weight, architecture, stiffness, porosity, degradability	• Lack of intrinsic bioactivity • Lack of nonlinear mechanical properties of native ECM	• Intestinal organoid	^56–59^
			• Liver organoid	^60,61^
			• Neural organoid	^62,63^
			• Bone marrow organoid	^64^
			• Pancreatic cancer organoid	^65^
PIC	• Thermoresponsive gelation • Fibrous, strain-stiffening architecture mimics native ECM • Tunable stiffness via molecular weight/concentration	• Poor biodegradability • Limited intrinsic bioactivity	• Mammary gland organoid	^72^
			• Liver organoid	^73^
PAAm	• Wide stiffness tunability • Stable over time and temperature	• Lack of intrinsic bioactivity	• Cardiovascular organoid	^77^
PVA	• Low toxicity • Tunable stiffness/degradability *via* physical or chemical crosslinking	• Lack of intrinsic bioactivity	• Pancreas organoid	^84^

Ref. indicates studies that reported organoid applications.

#### PEG hydrogel

PEG is a fully synthetic, hydrophilic polymer widely used in biomedical applications due to its biocompatibility, water solubility, and chemical inertness^44,45^. Its chemically defined nature, adjustable molecular weight, and controllable architecture (e.g., linear or multi-arm structures) offer key advantages over animal-derived ECMs for improving reproducibility in organoid culture^46^. To form a hydrogel, PEG macromers are typically functionalized with reactive end groups (e.g., acrylates or thiols) and crosslinked via chemical reactions such as photopolymerization, thiol–ene click chemistry, or radical polymerization^47–50^. This process yields a 3D network with tunable stiffness, porosity, and degradation profiles, allowing the creation of precisely defined microenvironments for organoid development. Compared to other synthetic hydrogels such as PIC, PAAm, and PVA, PEG offers exceptional commercial availability, structural versatility, and modular chemistry^51^. These features enable controlled incorporation of biochemical cues, such as integrin-binding peptides, matrix metalloproteinase (MMP)-sensitive crosslinkers, ECM-binding domains, or naturally derived components^52–55^. Such biofunctionalization strategies have expanded the applicability of PEG hydrogels across diverse organoid types, including intestinal^56–59^, liver^60,61^, neural^62,63^, bone marrow^64^, and pancreatic cancer organoids^65^.

**Fig. 2:**
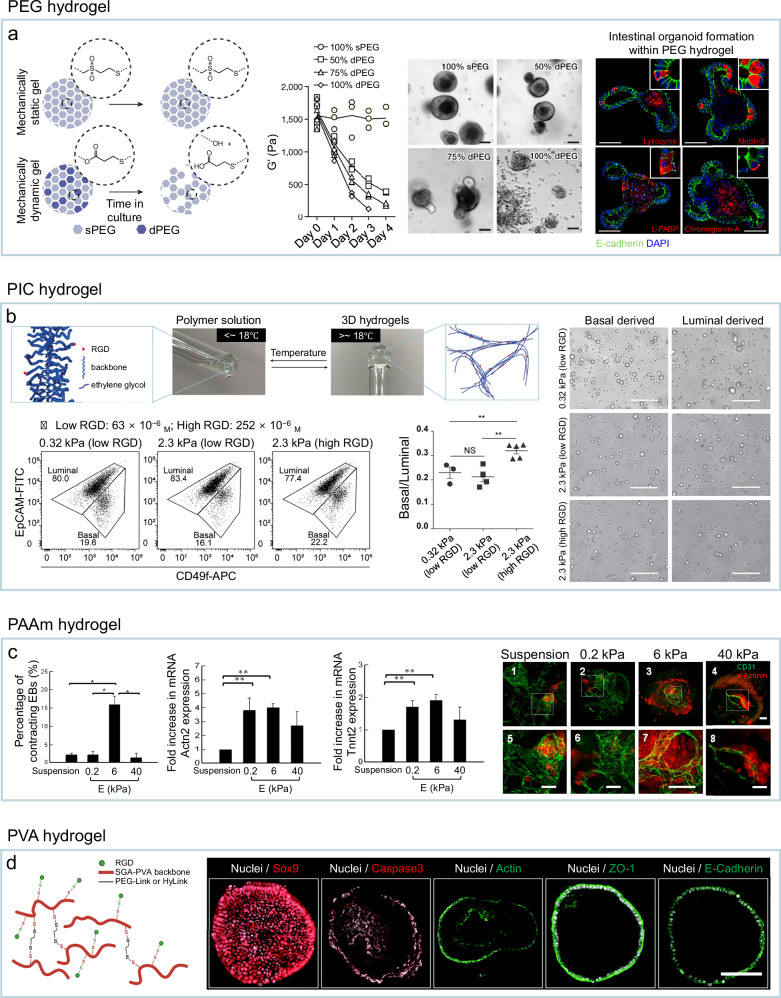
Organoid cultures in PEG, PIC, PAAm and PVA hydrogels. **a** PEG hydrogel engineered to switch from an expansion- to differentiation-permissive state, enabling controlled and reproducible intestinal organoid formation. Reproduced with permission^57^, Copyright 2016, Springer Nature. **b** PIC hydrogels (0.32 kPa, low RGD; 2.3 kPa, low/high RGD) allowing stiffness- and ligand-controlled regulation of basal-to-luminal ratios and morphologies in mammary gland organoids. Reproduced with permission^72^, Copyright 2020, John Wiley and Sons. **c** PAAm hydrogel matching native cardiac muscle stiffness, guiding vascularized cardiac-like organoid formation with reproducible outcomes. Reproduced with permission^77^, Copyright 2014, PLoS. **d** PVA hydrogel with RGD peptides and tunable crosslinkers for controlled, animal-free pancreas organoid culture. Reproduced with permission^84^, Copyright 2021, Royal Society of Chemistry.

One representative application is in intestinal organoid culture, where PEG hydrogels have been engineered to precisely control matrix composition, mechanics, and biochemical cues (Fig. 2a)^57^. Stage-specific requirements were identified: fibronectin-based adhesion and high matrix stiffness (≈1.3 kPa) promoted intestinal stem cell expansion via a YAP-dependent mechanism, whereas a soft matrix with laminin-based adhesion (≈190 Pa after softening) supported intestinal stem cell differentiation and organoid morphogenesis. Leveraging this controllability, mechanically dynamic PEG hydrogels were designed to shift from an expansion-optimized state (≈1.3 kPa) to a differentiation-permissive state (≈190 Pa), reproducibly generating mouse and human intestinal organoids across three independent experiments. Within this narrow mechanical window, organoids reliably developed crypt-villus-like architectures of similar overall dimensions (typically on the order of 100 µm) and displayed consistent branching patterns and lumen organization. The resulting structures uniformly expressed key functional markers—including lysozyme, mucin-2, chromogranin-A, and L-FABP—demonstrating reproducible morphogenesis and maturation across experiments.

A similar approach of tuning matrix composition and mechanics has been applied to bone marrow organoids, where PEG hydrogels were integrated with naturally derived hyaluronic acid (HA) and enzymatic transglutaminase (TG)-mediated crosslinking^64^. Using stiffness-matched formulations (storage modulus ≈ 240–320 Pa) and a 50/50 PEG/HA mixture with an onset of gelation around 60 s, this hybrid hydrogel system maintained stable mechanical and diffusional properties across independently prepared batches (*n* > 3), ensuring consistent physicochemical environments. Quantitative analyses further demonstrated enhanced maintenance and osteogenic differentiation of human bone marrow–derived mesenchymal stromal cells (ATP and ALP assays; upregulation of RUNX2, OSX, COL1A1, OPN, and OCN), alongside a ~3-fold expansion of CD34^+^ hematopoietic stem and progenitor cells (HSPCs/HSCs) compared to 2D controls. The reproducible cellular responses observed across multiple hydrogel formulations underscore the capacity of this defined system to support reliable bone marrow organoid generation.

#### PIC hydrogel

PIC hydrogels are thermoresponsive materials that form physically crosslinked networks upon heating above a sol–gel transition temperature, typically around 15 °C^66,67^. Once gelled, they maintain their structure under physiological temperatures such as 37 °C^68^. This mild and reversible gelation process enables easy handling and gentle cell encapsulation without the need for chemical crosslinkers, thereby minimizing cellular stress^69^. Their fibrous architecture and unique nonlinear mechanical behavior, such as strain-stiffening, closely mimic the physical characteristics of native ECMs, providing cells with physiologically relevant mechanical cues during organoid development^70,71^. A key advantage of PIC hydrogels lies in the precise tunability of their mechanical properties by adjusting the polymer molecular weight or concentration, allowing reproducible control over the biophysical environment^68^.

In one study, mammary gland organoids formed robustly and reproducibly in PIC hydrogels, maintaining > 98% cell viability over 7 days and consistent cystic organoid formation across repeated passages up to passage 10. When cultured in PIC hydrogels with stiffness values of 0.32 kPa or 2.3 kPa, combined with different RGD peptide concentrations, organoids exhibited distinct basal-to-luminal cell ratios (~0.2 and ~0.3, respectively, across multiple batches; *n* > 4) and morphological features, such as cystic versus branched structures, that correlated with matrix stiffness and ligand density (Fig. 2b)^72^. These results highlight the critical role of mechanical and biochemical cues in directing cell fate and tissue organization and demonstrate how PIC hydrogels can reproducibly control organoid architecture and composition across replicates. Extending this approach to liver organoid cultures, fine-tuning the stiffness of PIC hydrogels supplemented with a laminin-entactin complex enabled consistent proliferation dynamics and sustained differentiation potential. Organoids maintained the expression of stem cell markers (LGR5, SOX9, EPCAM, PROM1, AXIN2), proliferation markers (KI67, PCNA), and hepatocyte markers (ABCC2, ALB, CYP3A4), across multiple passages (at least 14 passages), underscoring the suitability of this system for long-term expansion^73^. Collectively, these examples demonstrate that PIC hydrogels provide a chemically defined, mechanically tunable platform capable of supporting robust and reproducible organoid development across diverse tissue types.

#### PAAm hydrogel

PAAm, a well-established synthetic hydrogel in mechanobiology, is increasingly recognized for its utility in organoid culture due to its exceptional mechanical tunability, enabling consistent performance^74,75^. By precisely adjusting the concentrations of acrylamide monomer and bis-acrylamide crosslinker, the stiffness of the hydrogel can be tuned across a wide physiological range, from sub-kilopascal to tens of kilopascals, without altering other matrix parameters^76^. This property allows systematic investigation of stiffness-dependent organoid differentiation and morphogenesis under highly reproducible conditions. Unlike biologically derived matrices or thermoresponsive materials such as PIC, PAAm hydrogels maintain stable mechanical properties over time and remain unaffected by temperature fluctuations, further enhancing experimental consistency^74^. In addition, the chemical nature of PAAm enables the decoupling of mechanical and biochemical cues, providing a defined platform for isolating mechanobiological effects.

For example, cardiovascular organoids have been generated by directing embryoid body (EB) differentiation on PAAm hydrogels with stiffness tuned to match that of native cardiac muscle (Fig. 2c)^77^. On 6 kPa gels—close to native cardiac muscle—the fraction of contracting EBs was ~7-fold higher than in suspension, 0.2 kPa, or 40 kPa conditions (*n* ≥ 100 EBs per group), while beating frequency did not differ significantly across groups. Immunostaining showed striated sarcomeric α-actinin (SAA)–positive cardiomyocyte sheets with CD31-positive vessel-like tubes most prominently at 6 kPa; SAA-positive area was roughly doubled versus the other conditions (*n* ≥ 20 EBs). Proliferation assays further indicated the highest proportion of EdU-positive cardiomyoblasts at 6 kPa, whereas EB diameters were comparable across conditions (*n* ≥ 50 EBs).

Although native PAAm lacks intrinsic bioactivity, functionalization with proteins or integration with bioactive hydrogels in interpenetrating polymer networks allows fine-tuning of the microenvironment for diverse organoid models while ensuring precise mechanical control.

#### PVA hydrogel

PVA is a water-soluble polymer with a high density of hydroxyl groups along its backbone, conferring strong hydrophilicity and high water uptake capacity^78^. It is widely used in biomedical applications due to its low toxicity, excellent biocompatibility, and chemical stability^79^. The availability of PVA in various molecular weights and degrees of hydrolysis further enhances its suitability for organoid culture systems where batch consistency and cytocompatibility are critical. PVA hydrogels can be formed via both physical and chemical crosslinking. Physically crosslinked PVA hydrogels are commonly prepared through repeated freeze–thaw cycles, which induce crystallite formation without chemical initiators and yielding elastic, cytocompatible gels with tunable stiffness depending on polymer concentration and processing conditions^80,81^. Alternatively, chemical crosslinking can be employed to enhance functionalization and achieve more precise mechanical control^82,83^.

A representative application is the development of PVA-based hydrogels functionalized with RGD peptides and tunable crosslinkers for fully defined, animal-free matrices for pancreas organoid culture (Fig. 2d)^84^. Incorporation of RGD peptides promoted cell adhesion, while the use of two distinct crosslinkers, non-degradable PEG-link and degradable HyLink, enabled independent control over stiffness and degradability. Among tested formulations, hydrogels with mechanical properties comparable to Matrigel supported efficient organoid formation (~80%) while maintaining structural stability, showing nuclear SOX9, polarized actin, E-cadherin, and ZO-1 patterns. Importantly, the chemically defined composition and tunable mechanics of PVA hydrogels enabled consistent organoid development across experiments (*n* = 3), underscoring their potential as a reproducible alternative to animal tissue-derived matrices.

### Strategies for controlling organoid morphogenesis

While synthetic hydrogels provide defined and tunable environments that mitigate batch-to-batch variability, achieving reproducible organoid morphogenesis additionally requires precise spatial regulation. Organoid formation is inherently stochastic, governed by self-organizing cellular programs that are highly sensitive to the spatial context established during early development^15^. Even under tightly controlled biochemical conditions, variability in initial cell positioning, local microenvironment, or mechanical boundaries can still lead to heterogeneous organoid architectures.

To address this, a range of engineering strategies has been developed to impose spatial control over organoid development and thereby reduce morphological variability. These approaches provide defined spatial cues that guide tissue morphogenesis, improving both the predictability and uniformity of the resulting structures. Broadly, spatial regulation strategies can be categorized into four primary mechanisms (Table 2): (1) external boundary confinement, which defines organoid size and shape through physical boundaries placed outside the forming organoids; (2) internal scaffold-guided organization, which introduces supporting structures within organoids to direct tissue orientation and organization; (3) spatially patterned modulation of matrix mechanics, which locally tunes mechanical properties of the matrix to influence morphogenetic processes; and (4) programmable cellular assembly, which precisely controls the spatial distribution and composition of cells to predetermine downstream organoid architecture.

**Table 2:** Engineering strategies for spatial regulation of organoid morphogenesis to enhance reproducibility

Strategy	Mechanism	Reproducibility	Implementation	Organoid type	Ref.
External boundary confinement	Physical boundaries restrict available space for aggregation and growth, controlling size and shape	Controls initial aggregate dimensions and reduces size variation, improving consistency in downstream morphology	Micropatterned substrate	• Pancreas	^85^
				• Intestinal	^86,87,94^
				• Lung	^88^
				• Brain	^89,90,95,96^
				• Kidney	^91,92^
				• Liver	^93^
			Microcapsule	• Intestinal	^97^
				• Brain	^99^
				• Hepatic	^98^
				• Islet	^100^
			Hanging drop	• Airway	^101^
				• Cancer	^102^
			Micropillar	• Brain	^103,104^
				• Liver	^105^
Internal scaffold-guided organization	Internal frameworks provide structural cues to guide shape, orientation, and patterning	Aligns early morphogenetic events and stabilizes complex tissue architectures	Suspended microfiber	• Brain	^108^
			Microfiber grid	• Brain	^109^
Spatially patterned modulation of matrix mechanics	Localized control of stiffness or viscoelasticity within matrix directs cell behavior and influences morphogenesis	Directs specific morphogenetic processes in a repeatable manner by delivering mechanical cues at defined sites	Photosensitive hydrogel with light-degradable crosslinker	• Intestinal	^94^
			Photopatterned modulation of crosslinking	• Intestinal	^110^
Programmable cellular assembly	Predefined composition, ratio, and spatial arrangement of progenitor populations regulate tissue patterning and organoid morphogenesis	Reduces stochastic variation in cell fate allocation and internal architecture	Inducible transcription factor–mediated multi-lineage differentiation	• Vascularized cortical	^111^
			DNA-based cellular patterning	• Kidney	^112^

Ref. indicates studies that reported organoid applications.

Each of these strategies enhances reproducibility by steering morphogenesis in a controlled manner, thereby improving the structural fidelity of organoids. The following subsections present representative examples of each approach and illustrate how spatial regulation has been leveraged to improve reproducibility across diverse organoid systems.

#### External boundary confinement

External boundary confinement is one of the most direct and widely adopted strategies for improving the reproducibility of organoid morphogenesis. By restricting the space available for cell aggregation and growth, physical boundaries reduce morphological variability and guide self-organization toward more uniform outcomes^16^.

**Fig. 3:**
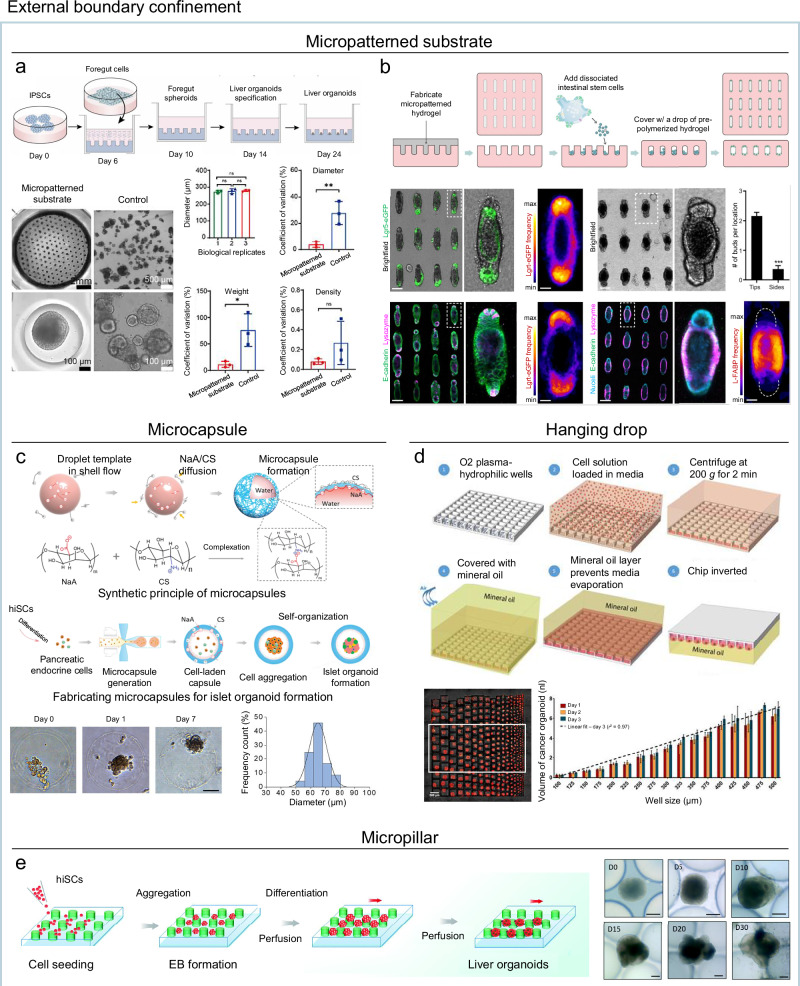
External boundary confinement strategies for reproducible organoid morphogenesis. **a** Micropatterned substrates for enhanced reproducibility of liver organoid production. Reproduced with permission^93^, Copyright 2022, IOP Publishing. **b** Micropatterned substrates for directed tissue organization in intestinal organoids. Reproduced with permission^94^, Copyright 2022, American Association for the Advancement of Science. **c** Microcapsules for spatial confinement of islet organoids. **d** Hanging drop culture for producing uniform sized-cancer organoids. Reproduced with permission^102^, Copyright 2020, John Wiley and Sons. **e** Micropillar for spatial confinement of liver organoids. Reproduced with permission^105^, Copyright 2018, Royal Society of Chemistry.

This strategy can be implemented through various engineered platforms, with micropatterned substrates representing one of the most widely used and versatile approaches. Micropatterned substrates can be fabricated from materials such as polydimethylsiloxane (PDMS), polystyrene (PS), or synthetic hydrogels like PEG, allowing precise control over geometry and spatial boundaries^18^. Microwell structures, in particular, physically confine organoid precursors, such as EBs or clusters of differentiated progenitors, within defined boundaries, regulating their subsequent expansion and enabling consistent size control. This approach has been successfully applied to various organoid types, including pancreas^85^, intestinal^86,87^, lung^88^, cerebral^89,90^, kidney^91,92^, and liver (Fig. 3a)^93^ models, producing uniform sizes across samples. In some cases, such confinement also improved internal structural uniformity. For example, intestinal organoids in PEG-based microwells showed consistent morphogenesis, with over 90% of organoids forming one or two crypt-like buds^86^, and kidney organoids in nanofibrous microwells exhibited more uniform nephron patterning and volumetric ratios of key structures (coefficient of variation (CV) < 10)^91^.

Beyond size control, geometry tuning in micropatterned substrates can direct tissue organization. For instance, intestinal stem cells seeded into elongated hydrogel cavities reproducibly formed epithelial structures mirroring the imposed shape, featuring an average of two crypt-like buds at curved ends and enterocytes populating flat central regions (Fig. 3b)^94^. Non-spherical geometries in neural organoids, such as triangular or star-shaped patterns, shifted developmental trajectories toward specific brain region identities^95^, while rectangular micropatterns enabled neural tube-like folding with over 90% structural fidelity^96^. In contrast, conventional cultures without geometric guidance produced highly variable morphologies, underscoring the critical role of spatial confinement in promoting reproducible organoid architecture.

An alternative approach is droplet-based encapsulation, often implemented within microfluidic systems. In this method, cells are enclosed in core–shell microcapsules with defined curvature and volume, enforcing a uniform spherical geometry. This strategy has been successfully applied to generate consistently sized intestinal^97^, hepatic^98^, brain^99^, and islet (Fig. 3c)^100^ organoids. Similar size-control effects can be achieved through hanging drop cultures for airway^101^ and cancer organoids (Fig. 3d)^102^, which allow aggregates to form in suspended droplets under gravity, and through micropillar arrays for brain^103,104^ and liver (Fig. 3e)^105^ organoids, where organoids are physically confined between pillars that restrict aggregate dimensions. While all these alternative techniques are highly effective for size control, they generally produce spherical or near-spherical aggregates, limiting morphogenetic versatility compared to micropatterned platforms with more complex geometries.

#### Internal scaffold-guided organization

In addition to strategies based on external boundary confinement, organoid morphogenesis can also be regulated through internal scaffolds that provide structural cues to guide organization. These scaffolds influence organoid shape, orientation, and spatial patterning during development, enabling controlled and reproducible morphogenesis. Unlike confinement strategies that physically enclose organoids within rigid boundaries, scaffold-guided approaches act as template-like frameworks that support self-organization while allowing more complex and tunable cell-environment interactions.

**Fig. 4:**
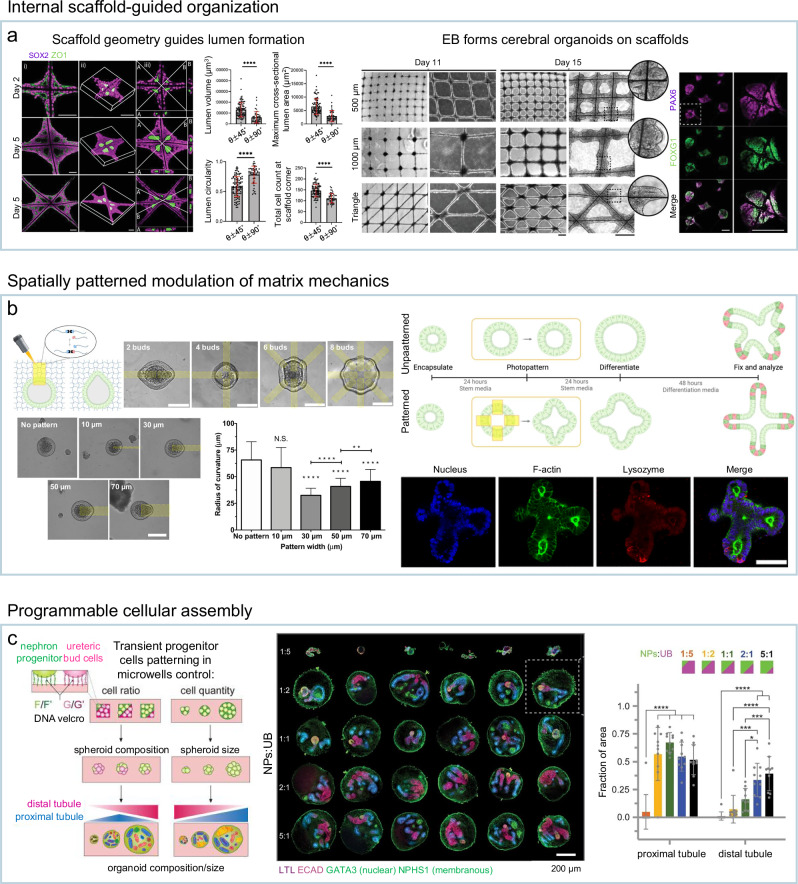
Examples of internal scaffold-guided organization, spatially patterned modulation of matrix mechanics, and programmable cellular assembly for reproducible organoid morphogenesis. **a** Microfiber grids with tunable mesh size and curvature controlling lumen positioning, size, and tissue organization in cerebral organoids. Reproduced with permission^109^, Copyright 2023, John Wiley and Sons. **b** Photopatterned modulation of hydrogel viscoelasticity to guide controlled budding events and epithelial curvature in intestinal organoids. Reproduced with permission^110^, Copyright 2022, American Association for the Advancement of Science. **c** DNA-based cellular patterning to control the initial composition and spatial arrangement of progenitor cells in kidney organoids. Reproduced with permission^112^, Copyright 2024, Elsevier.

Among various scaffold types, microfiber-based scaffolds have gained particular attention for their ability to direct organoid formation across multiple length scales. Microfibers, fabricated with natural or synthetic polymers, can be engineered with defined topographies, stiffness profiles, and geometries to influence cell alignment, aggregation, and differentiation at early developmental stages^106,107^. In neural organoid models, co-culturing human pluripotent stem cells (hPSCs) with suspended microfibers during EB formation guided early EB geometry, promoting the reproducible emergence of large telencephalic vesicles across samples^108^. Similarly, microfiber grids with tunable mesh dimensions have been used to control lumen positioning and size in cerebral organoids (Fig. 4a)^109^. Adjusting grid curvature and spacing yielded either discrete or interconnected morphologies, with greater structural consistency than conventional cultures.

#### Spatially patterned modulation of matrix mechanics

Spatially patterned modulation of matrix mechanics enables localized and dynamic control over the physical microenvironment of organoids, offering a distinct route to reproducible morphogenesis. Unlike external boundary confinement or internal scaffold-based approaches, this method delivers targeted mechanical cues, such as stiffness or viscoelasticity, within the matrix itself, allowing precise control over key morphogenetic processes, including symmetry breaking, budding, and epithelial folding. By confining mechanical modulation to defined regions, it minimizes variability arising from globally applied or uncontrolled mechanical environments.

One representative example used photosensitive PEG-based hydrogels with light-degradable crosslinkers and applied the materials to culture intestinal organoids^94^. Localized light exposure softened specific matrix regions, inducing budding events at predetermined sites. The resulting crypt-like structures consistently recapitulated native features, with Lgr5^+^ stem cells and Paneth cells positioned at bud tips and enterocytes in central domains. These results demonstrate that spatially defined matrix softening serve as a reproducible and sufficient cue for directed epithelial morphogenesis. In another study, photopatterned modulation of hydrogel crosslinking was employed to dynamically regulate matrix viscoelasticity during intestinal organoid development^110^. This spatiotemporal control reproduced crypt–villus architecture by inducing predictable epithelial curvature and activating mechanotransduction pathways such as YAP nuclear translocation (Fig. 4b). When applied before differentiation, the approach yielded highly consistent crypt formation across samples.

#### Programmable cellular assembly

Unlike previous strategies that impose spatial regulation through the organoid’s environment, programmable cellular assembly achieves control by directly defining the organization of the organoid itself. By precisely specifying the initial composition, ratio, and spatial positioning of distinct progenitor populations, this approach reduces stochastic variability from uncontrolled cell sorting and fate specification, thereby enhancing reproducibility at the level of intrinsic cellular organization.

Representative implementations include the simultaneous differentiation of human induced pluripotent stem cells (hiPSCs) into multiple lineages through inducible transcription factor overexpression, independent of culture medium composition^111^. This method generated vascularized cortical organoids in which endothelial and neuronal cells emerged in predictable domains after aggregation into multicore-shell embryoid bodies. Another example is DNA-based cellular patterning to assemble defined numbers and ratios of progenitor cells into organoid precursors (Fig. 4c)^112^. Using this approach, nephron progenitor (NP) and mosaic NP/ureteric bud (UB) organoids with tunable initial cell compositions were produced. An optimal NP-to-UB ratio consistently promoted proximal tubule formation, demonstrating how deliberate cellular configuration can reproducibly steer organoid development toward desired structural outcomes.

## Engineering strategies for enhancing scalability

Organoid-based applications often demand the production of large numbers of organoids. For example, high-throughput drug screening may require hundreds to tens of thousands of organoids to enable statistically robust comparisons across multiple compounds and dosage ranges. In tissue engineering and regenerative medicine, the scale required is even greater. Human adult-sized solid organs contain approximately 10–300 billion cells, with typical cellular densities ranging from 100 to 500 million cells per milliliter^113,114^. For instance, the human liver contains an estimated 160–360 billion cells^115,116^, while the heart comprises roughly 2–3 billion cardiomyocytes^117,118^. Given that a single organoid typically contains between 10^4^ and 10^6^ cells, reaching application-relevant cellular numbers would require on the order of thousands to tens of millions of organoids depending on the target organ.

However, conventional ECM matrix-based cultures support only a limited number of organoids per unit area, thereby constraining productivity. In response, substantial efforts have been directed toward developing platforms that can generate large numbers of organoids per batch. Yet as production volume increases, new bottlenecks emerge in associated processes that must also be scaled. For example, when thousands of organoids are cultured simultaneously, routine operations, such as media exchange, monitoring, and handling, become increasingly labor-intensive. Manual pipetting, still commonly used in many laboratories, is not suitable for managing such high-volume cultures efficiently.

Achieving truly scalable organoid production therefore requires a twofold approach: (1) development of technologies to increase organoid productivity, ensuring that large numbers of precursors and organoids can be produced efficiently; and (2) integration of supporting systems to enable high-throughput culture operations, ensuring that all subsequent steps can be performed at scale. The following sections present engineering strategies corresponding to each of these approaches, beginning with technologies for expanding organoid productivity, followed by systems that support large-scale culture.

### Strategies for increasing productivity of organoid precursors

Producing large numbers of organoids begins with generating a sufficient quantity of early-stage precursors, such as EBs or progenitor cell clusters. These structures are essential for downstream organoid formation and often represent a key bottleneck in scaling up production. Conventional methods that rely on spontaneous self-organization in matrix domes or standard well plates provide limited control over aggregation and typically yield only a small number of organoids per unit area. To overcome these limitations, engineering strategies to increase precursor productivity can be broadly classified into three categories: (1) increasing precursor density per unit area by partitioning cells into numerous micro-compartments for parallel formation, (2) enabling continuous precursor production through automated workflows that sustain high-throughput output, and (3) expanding culture volume to maintain and grow precursors in bulk quantities.

Each approach can be implemented using specific culture platforms tailored to the desired scale and application. Representative examples include microwell array platforms for high-density formation, droplet-based microfluidic systems for continuous production, and suspension bioreactors for large-volume expansion (Table 3). In the following subsections, we describe these platforms in detail and highlight their application to scalable organoid production.

**Table 3:** Platforms for increasing the productivity of organoid precursors

Platform	Principle	Advantage	Limitation	Representative productivity metrics	Ref
Microwell array	Cells settle into microwells, forming uniform aggregates in parallel within a compact area	• Enables high-density aggregate formation • Simple operation • Fixed spatial arrangement enables tracking	• Number of aggregates that can be generated is limited by surface area and dimensions of wells • High aggregate density may limit nutrient/oxygen	Up to ~1,000 organoids per 6-well; ~5 organoids per mm^2^	^86,92,119–123,126^
Droplet-based microfluidic system	Cells are encapsulated in monodisperse aqueous droplets as isolated microenvironments for aggregate formation	• Enables continuous, high-throughput production • Prevents fusion via physical isolation • Parallel operation of multiple chips allows throughput expansion	• Requires specialized microfluidic setup • Oil-based generation requires removal, causing cell loss, contamination	~1 aggregate per s; 10^2^–10^3^ organoids generated within 10 min; continuous 8–10 h runs possible	^97,98,127^
Suspension bioreactor	Dynamic mixing maintains aggregates in suspension, enabling large-volume culture	• Handles volumes from hundreds mL to liters • Provides uniform nutrient and oxygen distribution • Satisfies industry standard for large-scale bioprocessing	• Excessive shear stress can damage aggregates • Requires optimization of hydrodynamics	0.25–1 L working volume; ~10^4^–10^5^ organoids per batch	^139–143^

#### Microwell array

Microwell array platforms are a widely used approach for scaling up organoid production by enabling the parallel formation of large numbers of uniform cell aggregates within a compact area. Unlike conventional well plates, which typically yield only a limited number of organoids per well, microwell arrays incorporate hundreds to thousands of microwells into a single well, substantially increasing organoid yield per unit area.

**Fig. 5:**
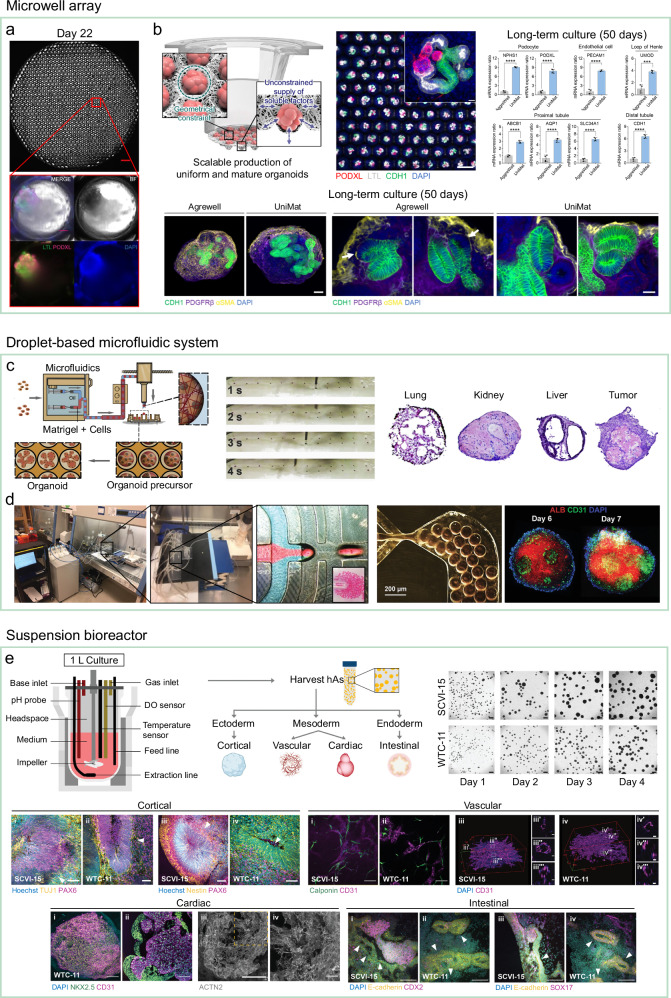
Examples of microwell arrays, droplet-based microfluidic systems and suspension bioreactors for increasing the productivity of organoid precursors. **a** Microwell array enabling scalable production of up to 1,000 kidney organoids. Reproduced with permission^119^, Copyright 2019, Springer Nature. **b** A permeable nanofibrous membrane–based microwell array (UniMat) enabling scalable production of uniform kidney organoids at ~5 organoids per mm^2^, while retaining functional markers and structural integrity for up to 50 days. Reproduced with permission^91^, Copyright 2024, Springer Nature. **c** Droplet-based microfluidic system enabling scalable production of highly uniform precursors, including lung, kidney, liver, and tumor models. Reproduced with permission^127^, Copyright 2020, Elsevier. **d** Long-term operation of droplet-based microfluidic system with multiple chips run in parallel to maximize throughput. Reproduced with permission^98^, Copyright 2023, John Wiley and Sons. **e** Stirred-tank bioreactor enabling scalable production of EBs, which can subsequently be differentiated into multiple organoid types. Reproduced with permission^143^, Copyright 2022, John Wiley and Sons.

A key advantage of microwell arrays is their operational simplicity. Without requiring complex microfluidic systems or specialized instrumentation, cells can be directly seeded into microwell-containing wells, where they spontaneously settle and form uniform aggregates. This straightforward workflow has been applied to the scalable production of multiple organoid types. For example, a single microwell-containing 6-well plate has been reported to yield up to 1000 kidney organoids (Fig. 5a)^119^, far exceeding the output of conventional formats, which typically yield only several dozen organoids per well. In addition, the fixed spatial arrangement of aggregates within microwells facilitates spatial indexing and long-term monitoring, making these platforms particularly suitable for high-throughput screening^86,92,120^. This contrasts with dynamic systems such as droplet microfluidics or suspension bioreactors, where constant motion complicates spatial tracking.

While standard microwell arrays in 12-, 24-, or 6-well plates already improve scalability, their capacity is ultimately limited by the physical dimensions of each well. Producing several thousand organoids in one batch often requires multiple wells, increasing manual workload from repeated cell seeding and media exchange. To address this, recent designs have expanded the microwell surface area beyond standard well dimensions. Some platforms now offer usable areas of ~50 cm^2^, enabling more than 100-fold higher yields compared to standard formats^121^. However, this expansion introduces challenges such as ensuring uniform cell distribution across the larger surface and developing efficient media-handling protocols that maintain aggregate quality.

Despite their scalability, microwell arrays present inherent limitations. High aggregate density within a confined area can limit nutrient and oxygen availability, impairing growth, maturation, or functionality. A common workaround is to use microwell arrays only for the initial aggregation phase, followed by transfer to larger suspension culture vessels for maturation^89,122^. Yet this introduces the risk of aggregate fusion, which can reduce size uniformity. One strategy to address this issue is to increase the viscosity of the culture medium; for example, adding biocompatible polysaccharides has been shown to improve yield and uniformity in cortical organoids^123^. Another approach is to enhance nutrient access without transferring aggregates. Microwell arrays fabricated on permeable substrates, such as porous polyethylene terephthalate (PET) membranes^124^, hydrogel sheets^125^, or nanofibrous membranes^126^, enable basal medium exchange and more efficient nutrient and oxygen diffusion, supporting long-term viability and scalability. For instance, a permeable nanofibrous membrane–based microwell array, termed UniMat, enabled scalable production of uniform and mature kidney organoids at a density of approximately five organoids per mm^2^, while retaining functional markers and structural integrity for up to 50 days, owing to the permeable properties of the nanofibrous membrane (Fig. 5b)^91^. In contrast, organoids cultured in conventional impermeable microwell arrays (AggreWell) exhibited structural collapse and functional decline, including fibrosis-like changes, during long-term culture. This contrast underscores the importance of mass transport and molecular diffusion in sustaining organoid viability and functional maturation. Efficient delivery of inductive signaling molecules is a key determinant of organoid quality and lineage fidelity, particularly in large-scale production where diffusional constraints limit the spatial distribution of morphogens. To ensure consistent differentiation, organoid engineering increasingly employs precisely timed and controllable supplementation of lineage-specific growth factors. Representative examples include Activin A, Wnt3A, EGF, FGF4, Noggin, R-spondin 1, and BMP4 for intestinal organoids; Activin A, FGF, TGF-α, HGF, BMP, R-spondin 1, nicotinamide, gastrin I, N-acetylcysteine, EGF, and Y-27632 for liver organoids; Activin A, Wnt, FGF10, BMP4, and TGFβ inhibitors for lung organoids; Activin A, BMP4, Wnt, and FGF for kidney organoids; TGFβ1, FGF2, Activin A, and BMP4 for cardiac organoids; Wnt, BMP4, and pluripotency factors (OCT4, SOX2, KLF4, C-MYC) for brain organoids; and EGF and BMP4 for skin organoids^20^. Accordingly, microwell arrays—and, more broadly, scalable organoid production systems—must be designed to ensure efficient and spatially uniform delivery of these factors, as precise control over morphogen distribution is essential for achieving reproducible differentiation outcomes across large-scale cultures.

#### Droplet-based microfluidic system

Droplet-based microfluidic systems enable continuous, high-throughput production of organoid precursors by compartmentalizing cells into thousands of monodisperse aqueous droplets, each serving as a self-contained microenvironment for aggregate formation. Unlike microwell-based platforms, which confine cells in fixed locations, droplet systems generate aggregates dynamically, typically at rates of around one per second using flow-focusing^98^ or T-junction geometries^127,128^. Cells are encapsulated within biocompatible hydrogel precursors such as alginate^99,129,130^, PEG-based materials^98,131^, or ECM-rich matrices like Matrigel^127,128,132,133^, which are subsequently polymerized to form stable microcapsules for long-term 3D culture.

A key advantage of this approach is its ability to operate continuously, enabling rapid and virtually unlimited production with minimal manual intervention. Physical isolation within droplets prevents aggregate fusion and promotes uniform growth, improving reproducibility. These features make droplet systems particularly attractive for industrial-scale production and for applications requiring strict batch-to-batch consistency. For example, one platform generated between 100 and 1000 highly uniform precursors, including lung, kidney, liver, and tumor models, in under 10 min (Fig. 5c)^127^. Other systems have been operated for 8–10 h continuously, with multiple chips operated in parallel to maximize throughput. In one such setup, a 25 mL working volume yielded approximately 25-fold more liver organoid precursors than a high-density microwell array of comparable footprint (Fig. 5d)^98^, underscoring the scalability of this method.

Despite these advantages, most high-throughput droplet systems rely on oil-based droplet generation, which offers excellent stability and precise size control but requires complete oil removal before downstream culture. Conventional removal methods, such as demulsification^134,135^, membrane absorption^136,137^, or filtration^138^, are often manual, introducing risks of cell loss, residual oil contamination, and reduced viability. Recent advances address this limitation by integrating on-chip oil removal modules, enabling direct transfer of cell-laden microcapsules from oil to aqueous media. This reduces handling steps, minimizes contamination risk, and improves process efficiency, as demonstrated in the scalable production of intestinal organoids^97^.

#### Suspension bioreactor

In contrast to static platforms, where culture volume is inherently constrained by container area and nutrient diffusion, suspension bioreactors enable large-volume expansion of organoid precursors by maintaining aggregates in dynamically mixed environments. When single cells are introduced into the agitated medium, hydrodynamic forces keep them in constant motion, promoting frequent cell–cell interactions and facilitating spontaneous aggregation without the need for physical compartmentalization. Continuous mixing prevents sedimentation and ensures uniform nutrient and oxygen distribution, reducing gradients that can impair viability and growth in large-scale cultures.

Several configurations have been adapted for organoid production, including spinner flasks^139,140^, rotating wall vessels^141,142^, and stirred-tank bioreactors^143^. These systems differ in mixing mechanics: spinner flasks use a centrally mounted stir bar to generate gentle bulk circulation, rotating wall vessels create a low-shear, simulated microgravity environment by rotating the entire culture chamber, and stirred-tank bioreactors employ impellers to deliver more controllable and scalable mixing. Despite these differences, all maintain aggregates in suspension over extended periods while accommodating large culture volumes suitable for scale-up. For example, human pluripotent stem cell-derived EBs cultured in a stirred-tank bioreactor with optimized impeller speed expanded over 4 days yielding a 16.6–20.4-fold increase in cell number, approximately 4 billion cells per 250 mL, scalable to 1 L, while retaining >94% expression of pluripotency markers (Fig. 5e)^140^. These expanded aggregates can subsequently be differentiated into various organoid types, including cardiac, vascular, cortical, and intestinal models.

The principal advantage of suspension bioreactors is their ability to handle working volumes ranging from hundreds of milliliters to several liters, offering substantial increases in total organoid output compared with microwell- or microfluidic-based formats. Owing to this capacity, they are widely regarded as the industry standard for large-scale bioprocessing. However, suspension cultures also present engineering challenges. Excessive shear stress from continuous mixing can damage delicate aggregates, whereas insufficient agitation can lead to clumping or fusion, reducing uniformity. To address these issues, recent bioreactor designs have incorporated optimized impeller geometries, intermittent mixing protocols, and low-shear hydrodynamic configurations to balance aggregate integrity with efficient mass transfer^144,145^.

### Strategies for supporting high-throughput culture operations

As organoid-generation platforms scale to produce thousands of units per batch, supporting culture operations must scale accordingly to avoid workflow bottlenecks. At large scale, manual execution of repetitive tasks quickly becomes impractical and labor-intensive, limiting the number of cultures a single operator can maintain. To address these constraints, automation strategies target two key aspects of large-scale workflows. The first is automated organoid culture, where dedicated platforms perform routine maintenance tasks, such as cell seeding, media exchange, environmental control, and real-time monitoring, across many culture units simultaneously. This approach ensures consistent growth conditions while drastically reducing hands-on time. The second is automated organoid handling, which reduces manual labor in transferring and manipulating organoids for downstream applications, thereby alleviating repetitive and time-consuming tasks for operators.

The following subsections detail representative technologies in each category, highlighting recent advances and their contributions to enabling truly scalable organoid workflows.

#### Automated organoid culture

As organoid production technologies advance toward scalable and high-throughput formats, automation systems have evolved in parallel to meet the increasing operational demands. These platforms have progressed from simple maintenance tools to multifunctional systems capable of dynamically controlling culture environments and performing complex workflows (Table 4).

**Table 4:** Categories of automated organoid culture platforms and their capabilities

Automation stage	Representative technologies	Key capabilities	Advantages	Ref
Early-stage automation (passive maintenance)	Automated microfluidic circuits	• Automated media exchange	• Reduces manual pipetting during media exchange	^146,147^
Multifunctional robotic platforms	Robotic liquid-handling	• Automated media exchange • Cell seeding • Drug exposure • Immunostaining assay	• Enables full workflow automation	^86,148,149^
Programmable microenvironment control	Pulse-width-modulated microfluidics with multi-well integration	• Automated media exchange • Real-time adjustment of soluble factor concentrations	• Reduces manual pipetting during media exchange • Enables programmable medium formulation	^150^
In-incubator imaging	Embedded imaging modules	• Automated media exchange • Real-time morphological analysis	• Reduces manual pipetting during media exchange • Eliminates need for plate removal, minimizing handling disturbances	^151^
AI-integrated platforms	Automated image analysis	• Automated media exchange • Real-time phenotypic assessment	• Reduces manual pipetting during media exchange • Label-free, non-destructive monitoring	^152,153^

Early automated organoid culture systems were developed to address a major labor-intensive bottleneck in large-scale workflows: media exchange. These early used simple fluid-handling mechanisms—e.g., automated microfluidic circuits^146,147^—to supply fresh medium and remove spent medium at scheduled intervals. By eliminating manual pipetting, they enabled the maintenance of many culture units in parallel, providing an important first step toward scalable operations. However, many of these early microfluidic systems required extensive tubing and valve networks (e.g., fluidic multiplexers) to achieve medium perfusion and withdrawal in each culture chamber. While effective in principle, such configurations increased the complexity of setup and maintenance, which limited their accessibility for routine use.

**Fig. 6:**
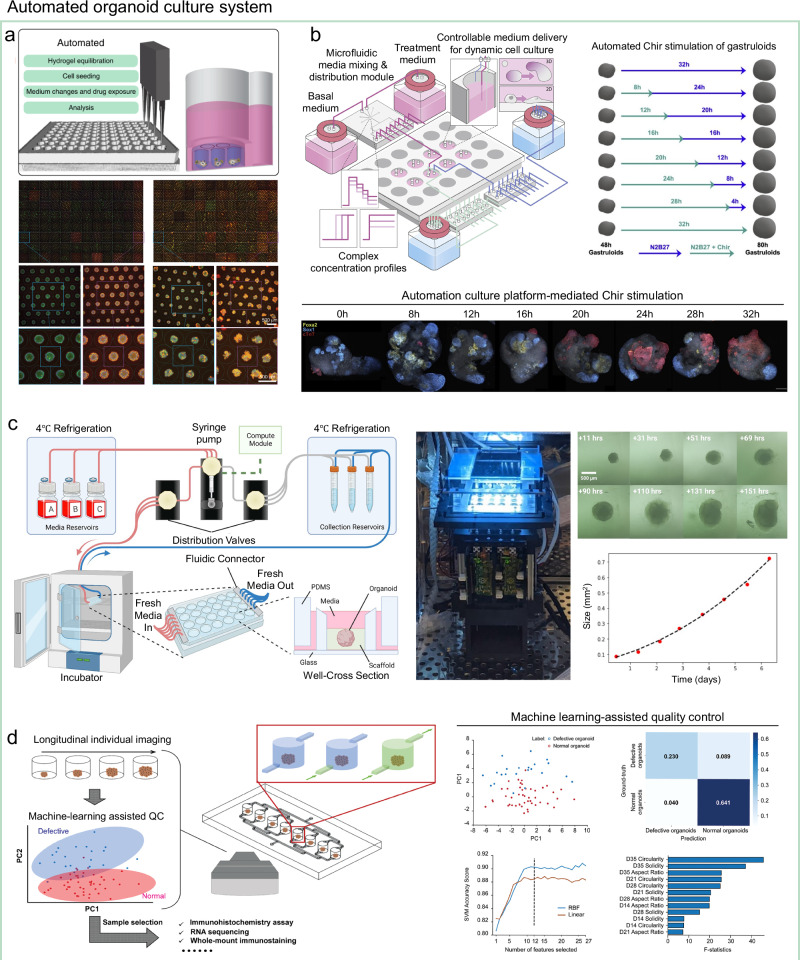
Examples of automated organoid culture. **a** Multifunctional robotic liquid-handling platform capable of full workflow automation. Reproduced with permission^86^, Copyright 2020, Springer Nature. **b** Automation using pulse-width modulation to deliver programmably changing medium compositions with precise temporal control. Reproduced with permission^150^, Copyright 2022, Elsevier. **c** In-incubator automated culture system integrating imaging hardware for real-time monitoring. Reproduced with permission^151^, Copyright 2022, Springer Nature. **d** Machine learning-integrated automation culture system for label-free quality control. Reproduced with permission^148^, Copyright 2024, John Wiley and Sons.

Robotic liquid-handling systems extend beyond media exchange. In addition to feeding, they automate cell seeding, controlled exposure to experimental conditions, and downstream processing steps required for analysis, including fixation, permeabilization, staining, and washing^86,148,149^. For example, one platform seeded intestinal organoids into microwell arrays, performed automated media exchanges for 6 days to support organoid expansion, carried out a three-day automated drug exposure protocol, and completed the entire fixation, permeabilization, staining, and washing sequence without manual intervention (Fig. 6a)^86^. Such multifunctional automation reduces labor demands and enables high-throughput experimentation with consistent timing and reproducibility across large culture sets. Nevertheless, because medium replacement in liquid-handling workflows can pose a risk of organoid loss, integration with structural or fluidic designs that minimize disturbance to aggregates will be essential to fully realize the benefits of automated systems. For instance, lateral perfusion schemes—exchanging medium via side channels rather than direct surface aspiration—reduce organoid displacement during repeated handling^86^. In addition, because open-well liquid-handling operations can expose cultures during medium replacement, careful sterility control remains important. Approaches such as operating within enclosed environments or incorporating in-incubator integration can help mitigate this risk, supporting more reliable long-term automation.

Subsequent developments extended beyond media replacement to incorporate real-time, programmable control of the culture microenvironment, shifting automation from passive maintenance to active, time-resolved modulation of soluble factor concentrations. For instance, microfluidic platforms using pulse-width modulation can continuously adjust medium composition to generate precisely timed concentration profiles, enabling systematic exploration of input–response relationships in organoid cultures (Fig. 6b)^150^. A notable example is a modular system integrating programmable microfluidic control with standard multi-well formats, automatically delivering dynamically changing medium compositions to multiple cultures in parallel, ideal for experiments requiring high temporal resolution over multi-day time courses.

More recent systems integrate environmental control with in-incubator monitoring to enable high-throughput operation without disturbing culture conditions. For example, in-incubator platforms embed imaging modules directly within the culture hardware to capture time-lapse images of organoid growth and morphology at regular intervals over extended periods (Fig. 6c)^151^. This eliminates plate removal during feeding, reduces handling, and allows uninterrupted long-term tracking of development. Building on these capabilities, next-generation systems incorporate automated image analysis and machine learning–based quality control^152,153^, enabling quantitative evaluation of organoid development in real time. Such platforms combine automated environmental regulation with label-free, high-resolution imaging to detect both gross morphological changes and subtle phenotypic variations in live organoids (Fig. 6d), allowing continuous, non-destructive assessment in their native 3D context. Automated feature extraction further supports objective classification of developmental states and pre-selection of organoids for downstream analyses.

#### Automated organoid handling

As organoid production scales up, downstream handling has emerged as a critical bottleneck. Manual transfer, positioning, and sorting are slow, labor-intensive, and prone to variability, making it difficult for downstream workflows to match the throughput of upstream generation platforms. Automated handling technologies address these challenges by enabling precise, repeatable, and high-throughput manipulation of individual organoids, thereby reducing operator workload and improving overall process efficiency (Table 5).

**Table 5:** Automated organoid handling methods

Method	Principle	Advantages	Limitations	Ref
Pick-and-Place	Aspirates organoids using negative pressure and deposits them at a target location by reversing the pressure	Straightforward to implement	• Low throughput • Mechanical stress	^154–158^
Drop-on-Demand	Encapsulates organoids in small, free-flying droplets delivered to specific locations	Non-contact handling	• Throughput depending on proportion of desired organoids • Risk of nozzle clogging	^161,162^
Magnetic guidance	Uses magnetic fields to manipulate magnetically responsive organoids	Gentle handling	• Requires magnetically responsive samples	^164,165^
Pick-Flow-Drop	Combines Pick-and-Place aspiration with precise droplet dispensing	Selective handling of heterogeneous samples	• May inherit limitations of both methods if not optimized	^166^

**Fig. 7:**
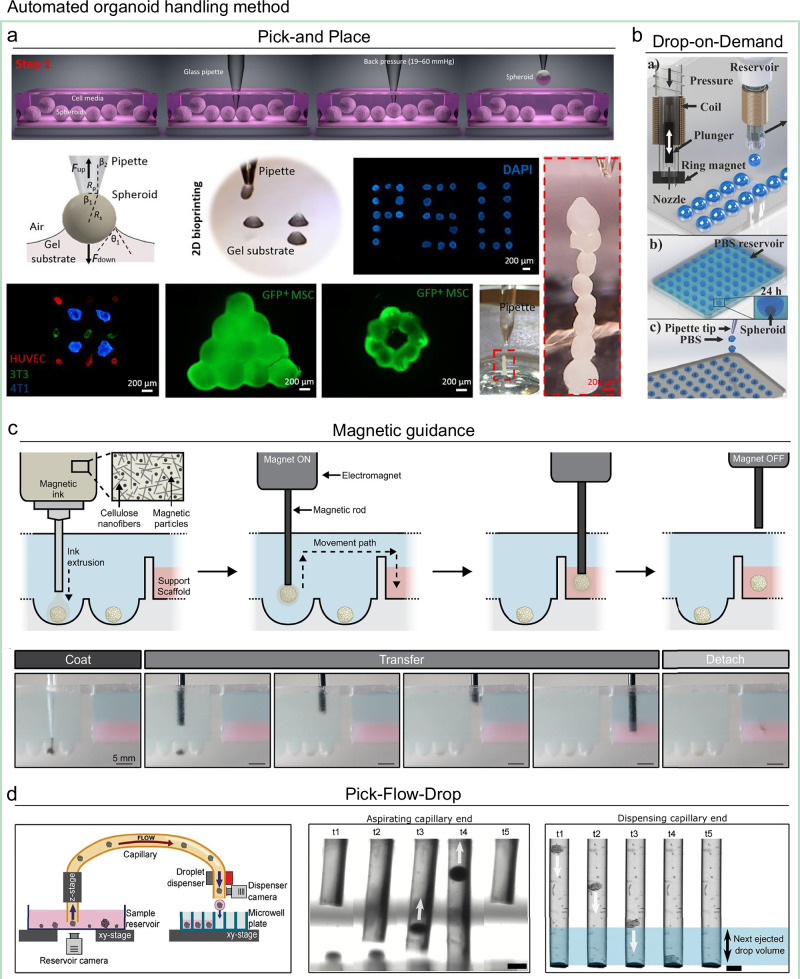
Examples of automated organoid handling technologies. **a** Pick-and-Place: aspiration and deposition of organoids using negative and positive pressure. Reproduced with permission^155^, Copyright 2020, American Association for the Advancement of Science. **b** Drop-on-Demand: dispensing organoids in free-flying droplets. Reproduced with permission^162^, Copyright 2022, IOP Publishing. **c** Magnetic guidance: gentle positioning of magnetically responsive organoids. Reproduced with permission^165^, Copyright 2023, Springer Nature. **d** Pick-Flow-Drop: hybrid of Pick-and-Place and Drop-on-Demand for selective handling. Reproduced with permission^166^, Copyright 2024, John Wiley and Sons.

One well-established approach is the Pick-and-Place method (Fig. 7a), in which organoids are aspirated into a pipette tip or fine capillary using negative pressure and then deposited at a target location by reversing the pressure^154–158^. While straightforward and broadly applicable across organoid types, its scalability is limited by relatively low throughput, often only a few organoids per min, and involves relatively large transfer volumes and mechanical stress during aspiration^159,160^. Furthermore, closely spaced organoids cannot be easily handled individually, requiring upstream culture conditions to be optimized for organoid spacing and density in reservoirs. An alternative is non-contact dispensing—Drop-on-Demand (Fig. 7b)—in which organoids are encapsulated in small, free-flying droplets and delivered directly to specific target locations^161,162^. This method can integrate in-line property detection, such as size measurement, near the dispensing nozzle, enabling selective placement based on predefined criteria. However, its throughput and efficiency depend strongly on the fraction of desired organoids in the sample, and nozzle clogging remains a common challenge in dense or impure suspensions^163^.

Other methods exploit physical forces for manipulation, such as magnetic guidance (Fig. 7c)^164,165^, which can provide gentle handling but are constrained by the need for magnetically responsive samples, limiting their generalizability in large-scale workflows. Hybrid strategies have also emerged to combine the strengths of multiple approaches. One example is the Pick-Flow-Drop method, which merges aspects of Pick-and-Place and Drop-on-Demand (Fig. 7d)^166^. In this approach, organoids are first aspirated from a reservoir and guided through a capillary, then precisely dispensed at target locations. This design allows selective handling of heterogeneous samples, reduces sample loss, increases throughput, and maintains compatibility with various organoid types and substrates.

Although automated handling technologies remain in the early stages of industrial-scale adoption, their rapid evolution positions them as a key enabler of fully integrated, end-to-end organoid production pipelines, bridging the gap between high-volume generation and equally scalable downstream processing. Moreover, advanced organoid-handling methods are not only essential for scalable processing but also provide a technical foundation for manufacturing advanced multi-lineage organoid tissue, commonly referred to as assembloids^167,168^. The automated positioning systems enable controlled fusion of distinct organoid types or the incorporation of additional cell populations, facilitating spatially organized co-cultures that recapitulate inter-tissue interactions. For example, assembloid models now incorporate immune components to recreate immune–epithelial interactions and stromal compartments to model epithelial–stromal crosstalk in gastrointestinal assembloids^169^. Similarly, neuronal or neural-crest-derived elements are integrated into brain-region organoids to form functional neural circuits in assembloids^170^. By enabling precise alignment, minimal mechanical stress, and reproducible assembly of heterogeneous tissue units, these advanced organoid-handling technologies thus bridge conventional culture systems with next-generation assembloid manufacturing. Collectively, these advances highlight that the ability to physically and functionally merge organoids with immune, stromal, or neuronal partners will be key to producing physiologically relevant composite tissues and to modeling systemic interactions in vitro.

## Applications accelerated by reproducible and scalable production

Recent engineering advances that enhance both reproducibility and scalability are helping to overcome the inherent limitations of organoid technologies, thereby enabling more precise, high-throughput drug testing and advancing regenerative medicine applications, as discussed in the following sections.

### Toward organoid-based precision translational applications

The translation of organoid research into precision medicine critically depends on producing large numbers of physiologically relevant organoids with high reproducibility and scalability—qualities that ensure observed phenotypic or molecular differences in downstream assays reflect genuine biological variation rather than technical noise. Recent engineering advances have begun to overcome the bottlenecks of conventional organoid culture, enabling more standardized and statistically robust drug testing.

**Fig. 8:**
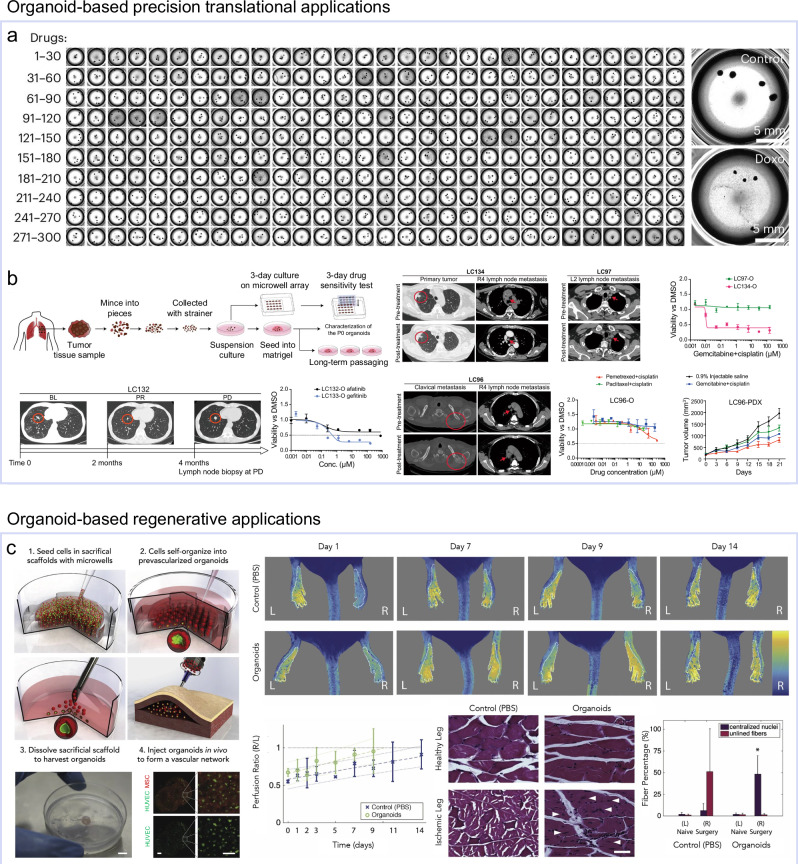
Reproducible and scalable organoids for translational and regenerative applications. **a** High-throughput neurotoxicity screening of human cortical organoids, enabling testing of ~300 FDA-approved compounds across > 2,400 organoids. Reproduced with permission^171^, Copyright 2025, Springer Nature. **b** Patient-derived lung cancer organoids providing clinically meaningful drug-response profiles consistent with xenografts models, mutation analyses, and clinical outcomes. **c** Vascularized organoids-based macroscale tissue construct promoting rapid in vivo vascularization in mice.

Microwell arrays have been especially effective in accelerating precision translational applications by generating large batches of highly uniform organoids. For example, human cortical organoids produced using microwell arrays with consistent size and morphology enabled standardized screening of ~300 FDA-approved compounds across more than 2,400 organoids, supporting high-throughput neurotoxicity testing (Fig. 8a)^123^. Similarly, a nanofibrous microwell array (UniMat) improved both size uniformity and tissue maturation of kidney organoids, reducing variation to a CV < 10% and enabling standardized polycystic kidney disease (PKD) disease modeling suitable for statistical drug testing^91^. Other microwell arrays demonstrated rapid clinical utility; producing over 100 lung cancer organoids within one week allowed acquisition of clinically meaningful drug response profiles in agreement with patient-derived xenografts, tumor mutation profiles, and clinical outcomes (Fig. 8b)^171^. This rapid turnaround offers the potential to shorten the biopsy-to-decision timeline for personalized oncology. Likewise, patient-derived pancreatic cancer organoids generated on microwell arrays maintained <5% size variation and preserved native genotype and phenotype, enabling chemotherapeutic and immunotherapeutic testing and demonstrating potential as a companion diagnostic tool^172^.

Integration of automation technologies with microwell arrays shows strong potential to advance translational organoid applications. A hydrogel microwell array, paired with a robotic handling system, enables fully automated derivation of intestinal organoids^86^. Uniform microwell geometry and controlled seeding density minimize size variability, while positioning all organoids in a single focal plane improves imaging throughput and analytical accuracy. This setup can generate thousands of uniformly sized intestinal organoids suitable for high-content phenotypic screening. Similarly, a liquid-handling pipeline that automates the entire kidney organoid differentiation protocol produces PKD models at scale^148^. This system delivers pharmaceutical-grade scalability and throughput capacity, supporting large-scale, high-content screening.

Collectively, these examples highlight how engineering-driven control over organoid production can transform organoids into reliable, high-throughput platforms for precision translational medicine, enabling both accelerated drug discovery and personalized therapeutic decision-making.

### Toward organoid-based regenerative applications

Although examples of regenerative medicine directly integrating engineering technologies for reproducible or scalable organoid production remain limited, a number of proof-of-concept studies have demonstrated that organoids hold substantial potential for tissue repair and functional restoration, as introduced in prior reviews^173–176^. However, most regenerative organoid applications remain at the laboratory scale, and achieving clinically relevant manufacturing under full GMP compliance will require closing residual engineering gaps.

Recent advances in organoid engineering for reproducible and scalable production are beginning to address these limitations. A fully defined, mechano-modulatory PEG hydrogel platform represents a notable innovation^61^. This system enables reproducible generation of liver organoids directly from patient biopsies, exhibiting key hepatic functions, including albumin secretion, urea production, glycogen storage, and low-density lipoprotein (LDL) uptake. By eliminating animal-derived matrices and allowing precise control over matrix mechanical properties, this approach overcomes major hurdles to GMP-compliant liver organoid manufacturing and provides a clinically translatable foundation for applications such as autologous hepatocyte transplantation.

Microwell array systems also show strong potential. A sacrificial hydrogel-based microwell array can generate tens of thousands of vascularized organoids within a single 60-mm dish, achieving high reproducibility in size and functionality (Fig. 8c)^177^. Approximately 30,000 such organoids were assembled into a macroscale tissue construct (~1 cm) for implantation, which promoted rapid in vivo vascularization in mice. This strategy highlights the promise of organoid-based tissue regeneration, particularly when coupled with scalable organoid production technologies. Similarly, stirred-tank bioreactors have been employed to produce large numbers of iPSC aggregates at clinically relevant scales, paving the way for patient-specific regenerative therapies, including autologous tissue grafts and organ-scale bioengineered implants^140^.

Another critical step toward clinically relevant regenerative applications of organoids is the establishment of functional vascular networks within macroscale tissue constructs. Because diffusion limits of oxygen, nutrients, and metabolites restrict the viable thickness of avascular organoids to approximately 200–300 µm, vascularization technologies are indispensable for maintaining cell viability and function in large-scale tissues. Recent advances have introduced diverse strategies that enable organoids to either self-generate intrinsic vascular networks or establish functional anastomoses with surrounding host vasculature. These include perfusion- and flow-enhanced endothelialization of organoids^119,178^; co-culture of endothelial and mesenchymal progenitors within self-assembling hydrogels to promote capillary ingrowth^177^; and scaffold-guided spatial patterning to preform perfusable vascular channels^179,180^. Such approaches collectively demonstrate that vascular integration markedly enhances the maturation, oxygenation, and functional stability of organoid-derived tissues in vitro^181,182^. For example, cardiac organoid models incorporating endothelialization exhibit improved electrophysiological function, contractile performance, and tissue organization, while perfusable organoid-on-chip hybrids integrating microvascular channels or endothelialized circuits provide controlled flow and shear stress that sustain long-term functionality and viability^24,183,184^. Altogether, organoid vascularization technologies are expected to play a pivotal role in enabling construction of centimeter-scale, vascularized organoid tissues capable of supporting true regenerative transplantation in vivo.

Together, these developments illustrate how engineering-driven advances in reproducibility and scalability can help bridge the gap between proof-of-concept regenerative organoid research and clinically deployable therapies. In this context, regenerative applications will likely require multi-variety manufacturing of organoids tailored to diverse patient needs and disease indications, further emphasizing the importance of flexible yet standardized production platforms. Ultimately, such innovations may accelerate the translation of organoids into routine medical practice.

## Conclusions and future perspectives

Organoid technology has evolved from an experimental laboratory model into a promising platform with substantial potential in drug discovery, disease modeling, and regenerative medicine. Despite its advances, organoid technology still faces two key challenges: reproducibility and scalability—the reliance on biologically variable materials, such as animal-derived ECMs, combined with the stochastic nature of morphogenetic processes, causes significant batch-to-batch and inter-sample variability, while conventional low-productivity culture methods and labor-intensive, low-throughput workflows constrain scalability, creating bottlenecks in applications that require large, uniform organoid populations.

Recent advances in engineering technologies have begun to reframe organoid culture as a controllable manufacturing process rather than a purely biological phenomenon. Defined synthetic hydrogels have addressed matrix-related variability. Spatial regulation approaches that constrain morphogenesis also improve uniformity. In parallel, high-throughput production platforms have substantially increased production yield, while automated culture and handling systems have reduced manual labor and improved process consistency. Collectively, these diverse engineering strategies can be organized into a conceptual framework of best practices for organoid manufacturing, where each technology contributes distinct advantages, limitations, and optimal conditions for implementation (Table 6). These advances mark a decisive shift from artisanal organoid culture toward standardized, industrializable workflows.

**Table 6:** Summary of best practices across major engineering technologies for organoid manufacturing

Engineering technology	Key attributes	Advantages	Limitations	Best practice (recommended use)
Synthetic hydrogels	Chemically defined ECM analogs with tunable stiffness, degradability, and ligand presentation	Minimize batch variability and provide precise tunability of matrix properties	• Reduced biochemical complexity • Lineage-dependent optimization required	• Used as a reproducible ECM substitute such as ECM-coated well plate or hydrogel embedding culture
Spatial regulation strategies	Engineered constraints and guidance mechanisms for spatial control of morphogenesis	Improve morphological fidelity and tunability by directing geometry, polarity, and organization	• Complex fabrication • Limited scalability and material compatibility depending on strategy	• Used to define geometry or impose morphogenetic patterning cues on organoid precursors
High-productivity platforms/systems	High-density, continuous, or dynamic organoid production platforms /systems	Improve production efficiency and enable large-quantity organoid culture	• Diffusion or shear-stress limitations depending on platform/system design	• Used for large-batch formation and expansion of organoid precursors
Automated systems	Instrument- and automation-based systems minimizing operator manual workload throughout the culture process	Reduce hands-on labor and streamline culture workflows across production stages	• High setup cost • Protocol-specific customization required	• Used to automate manual operations such as media exchange, real-time monitoring, and organoid handling

**Fig. 9:**
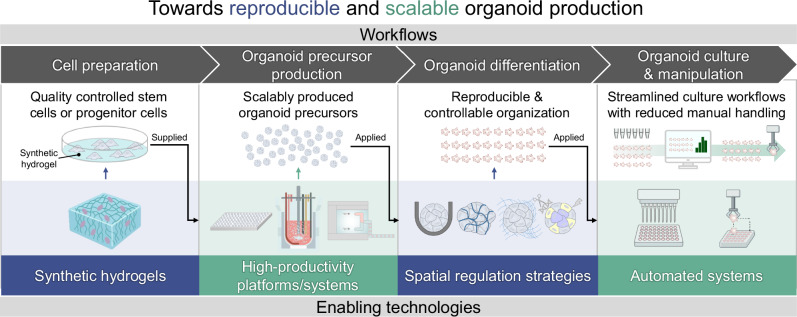
Schematic showing how engineering technologies map to specific stages of the organoid production workflow.

However, most of these technologies have been developed in isolation, often optimized for specific organoid types, production stages, or experimental goals, rather than as components of an integrated manufacturing pipeline. Consequently, while each approach individually improves certain aspects of reproducibility or scalability, the absence of interoperability limits their overall impact when moving toward industrial or clinical deployment. Protocols and hardware optimized within one context may not be directly transferable to another without substantial re-optimization, custom interfacing, or workflow redesign. Such mismatches create technical and operational barriers that not only slow the scaling process but also introduce additional sources of variability. Integrating these diverse engineering innovations—synthetic hydrogels, spatial regulation strategies, high-productivity platforms/systems, and automated systems—within corresponding stages of cell preparation, organoid precursor production, differentiation, and culture and manipulation could bridge these gaps (Fig. 9). Establishing such interoperable links across the workflow would enable cohesive, closed-loop manufacturing pipelines capable of delivering reproducible and scalable organoid production across diverse applications.

Additionally, emerging artificial intelligence (AI) technologies are increasingly being incorporated into organoid research^185–187^. To date, AI has primarily been applied to analytical tasks such as high-throughput image-based phenotyping, automated morphological classification, and integration of multi-omics datasets^188–193^. These applications have accelerated data interpretation and reduced operator-dependent bias; however, their role in organoid manufacturing has been limited. Expanding AI into the production process could substantially improve both reproducibility and scalability. For example, AI-driven image analysis pipelines could be embedded into culture systems to provide real-time monitoring of the differentiation process, enabling continuous cell quality control and early detection of deviations that may compromise reproducibility. In current practice, differentiation conditions, such as factor concentrations and timing, are often determined empirically based on individual researcher experience, which can lead to variability. Training AI models on large datasets of past differentiation outcomes could enable predictive optimization of these conditions, thereby standardizing protocols and enhancing both reproducibility and productivity. Furthermore, because optimal differentiation parameters can vary across stem cell lines and batches, AI could detect and adapt to these variations, ensuring consistent results across diverse input sources. Cell source variation, including genetic background, epigenetic memory, and donor-to-donor heterogeneity, remains a major contributor to experimental variability, and integrating AI-driven adaptation could help mitigate its impact. Coupled with automated culture platforms, such algorithms could dynamically adjust feeding regimens, biochemical gradients, or mechanical cues in response to continuous imaging or biosensor feedback, creating adaptive, self-correcting manufacturing pipelines.

Beyond image analytics, the integration of AI and multi-omics is rapidly emerging as a powerful framework for quantitative quality control and mechanistic insight in organoid research^185,194^. Single-cell RNA sequencing (scRNA-seq), spatial transcriptomics, proteomics, and metabolomics now enable comprehensive profiling of lineage composition and functional maturation. When coupled with AI-driven data integration methods—such as deep generative models and graph neural networks—these datasets can reveal hidden regulatory relationships, correct batch-specific biases, and predict differentiation trajectories across large organoid cohorts^195^. Embedding such integrative analyses directly into production pipelines could establish real-time molecular quality control, linking morphological phenotypes with underlying transcriptomic and metabolic states. This convergence of imaging, multi-omics, and machine intelligence will be crucial for developing adaptive, self-optimizing organoid manufacturing systems that ensure reproducibility at both phenotypic and molecular levels.

Moreover, integrating scientific knowledge and engineering techniques that enable a deeper understanding and precise regulation of tissue developmental processes and morphogenesis could play a pivotal role in realizing the full spectrum of organoid-based applications^179,180,196–198^. Such integration would not only allow researchers to manipulate cellular behaviors at the molecular and microenvironmental levels but also facilitate the recreation of complex, hierarchical tissue and organ architectures in vitro. In particular, when combined with scalable and reproducible organoid production technologies, these engineering approaches could empower organoids to faithfully recapitulate tissue- and organ-level structures, physiological functions, and dynamic responses to environmental cues. This convergence may ultimately lead to the creation of next-generation artificial organ models with predictive clinical relevance, high-throughput compatibility for drug discovery, and disease modeling. Moreover, as biofabrication, biomaterial design, and vascularization strategies continue to evolve, these organoid systems could be further adapted into implantable constructs, opening avenues for regenerative medicine applications where functional tissue replacement is feasible, safe, and personalized.

In conclusion, recent engineering advances have brought about a new era in organoid technology, transforming it from an artisanal laboratory method into a more standardized and high-throughput platform. By addressing current limitations and embracing emerging innovations, the field can progress even further toward realizing the vision of truly reproducible and scalable organoid manufacturing. Moving forward, realizing practical clinical and industrial applications will require not only scalable and reproducible production, as emphasized throughout this review, but also attention to critical factors such as cost, throughput, and ethical–clinical governance. From a commercialization standpoint, reducing dependence on biologically variable and costly matrices like Matrigel through defined synthetic alternatives, implementing automated and closed-loop bioreactor systems, and embedding in-line quality control are key to improving cost efficiency and production yield while maintaining reproducibility and GMP compliance. At the same time, the use of patient-derived organoids requires robust ethical and clinical governance as the field advances toward biobanking, drug testing, and eventual commercialization. Key priorities include transparent donor consent and data use, equitable governance of benefit-sharing and commercialization, and ensuring compatibility with clinical-grade workflows to support responsible clinical translation. Together, these efforts will pave the way toward the next generation of organoid technologies capable of bridging research, industry, and clinical practice.

## Data Availability

No datasets were generated or analysed during the current study.
